# A new silver nanorod SPR probe for detection of trace benzoyl peroxide

**DOI:** 10.1038/srep05323

**Published:** 2014-06-17

**Authors:** Zhiliang Jiang, Guiqing Wen, Yanghe Luo, Xinghui Zhang, Qingye Liu, Aihui Liang

**Affiliations:** 1 Key Laboratory of Ecology of Rare and Endangered Species and Environmental Protection of Ministry Education, Guangxi Normal University, Guilin 541004, China; 2 These authors contributed equally to this work.

## Abstract

The stable silver nanorod (AgNR) sol in red was prepared by the two-step procedure of NaBH_4_-H_2_O_2_ and citrate heating reduction. The AgNR had a transverse and a longitudinal surface plasmon resonance (SPR) absorption peak at 338 nm and 480 nm. Meanwhile, two transverse and longitudinal SPR Rayleigh scattering (SPR-RS) peaks at 340 nm and 500 nm were observed firstly using common fluorescence spectrometer. The SPR absorption, RS, surface enhanced Raman scattering (SERS) and electron microscope technology were used to study the formation mechanism of red silver nanorods and the SERS enhancement mechanism of nano-aggregation. The AgNR-BPO SPR absorption and AgNR-NaCl-BPO SPR-RS analytical systems were studied to develop two new simple, rapid, and low-cost SPR methods for the detection of trace BPO.

Precious metal nanoparticles, especially nanogold and nanosilver have become research focus in many fields such as physics, chemistry, materials and sensing since they have novel physicochemical properties and good stability^1^,^2^. Compared with nanogold, nanosilver (AgNP) has advantages of low-cost, higher molar extinction coefficient^3^ and more excellent optical properties such as the AgNP aggregates being of low molar extinction coefficient and strong SERS effects, it provide the foundation for their applications^4^,^5^,^6^,^7^,^8^. SPR characteristics are quite remarkable among the optical properties of nanoparticles such as AuNP and AgNP. Under the irradiation of external light field, nanoparticles would generate surface plasma, that is, collective electron excitation^9^. Nanoparticles keep still when electrons left the equilibrium position because the quality of surface electron is much smaller than that of the nanoparticles and the surface charge has not been offset. Electron cloud will oscillate in its equilibrium position due to the effect of restoring force, and the frequency was depended on the electron density, effective electron mass, the shape and size of charge distribution and environment. Spherical nanogold or nanosilver had only one SPR absorption peak, while nanorod had two SPR absorption peaks, which one was transverse and the other was longitudinal, and triangular nanodisc had three SPR absorption peaks. The characteristics constitute the basis of nanogold and nanosilver in the biological sensing and imaging^10^,^11^,^12^. At present, the synthesis of stable nanogold/nanosilver, especially nanorods has attracted much attention^13^,^14^,^15^. Gold nanorods are commonly prepared by the seeds method^13^,^14^, which need a high concentration of cationic surfactant and purification to remove the excess of surfactant, this made its applications were limited. Compared with the gold nanorods, the synthesis of silver nanorods is less, it mainly include the reduction and template methods, which can be used for the preparation of silver nanorods sol and silver nanorod arrays^16^,^17^,^18^,^19^,^20^. In the presence of cationic surfactant CTAB and gold nanorods as seed, Au core-Ag shell nanorods sol can be prepared with ascorbic acid reducing AgNO_3_, but they need centrifugal separation^21^. Therefore, to explore a simple, rapid, and environmentally friendly method for synthesis of stable silver or gold nanorods has very significance.

Benzoyl peroxide (BPO), once used as flour additives to improve the color and lustre of wheat flour and corn starch, has oxidation to vitamin and carotene, and result in the destruction of the flour nutrients. Excessive intake of BPO from wheat flour has a bad effect on the liver. Short-term excessive intake can lead to symptoms such as nausea, dizziness and neurasthenia, while long-term excessive consumption will seriously damage the liver^22^. In 2011, seven departments including ministry of health officially announced that BPO is prohibited to add into flour in China. So far, the analytical methods for BPO in flour mainly include spectroscopy, chromatography, mass spectrometry and electrochemical method^23^,^24^,^25^,^26^,^27^. RRS, using synchronous scanning technology by common fluorescence spectrometer, has the characteristics of simple and sensitive, and was used in biochemical analysis combining with nanogold or nanosilver^7^,^28^,^29^,^30^. As we know, there have no reports about the preparation of red silver nanorods using the blue triangle nanosilver sol as precursor by heating and its plasmon resonance spectrometry for detection of BPO yet. In this article, the stable AgNR sol was prepared easily, the AgNR-BPO-NaCl system has been studied in details by SPR absorption, RRS, SERS, electron microscopy and other technology, and two simple, rapid and sensitive methods have been developed for detection of BPO.

## Results

### Transmission electron microscope (TEM)

**Figure 1:**
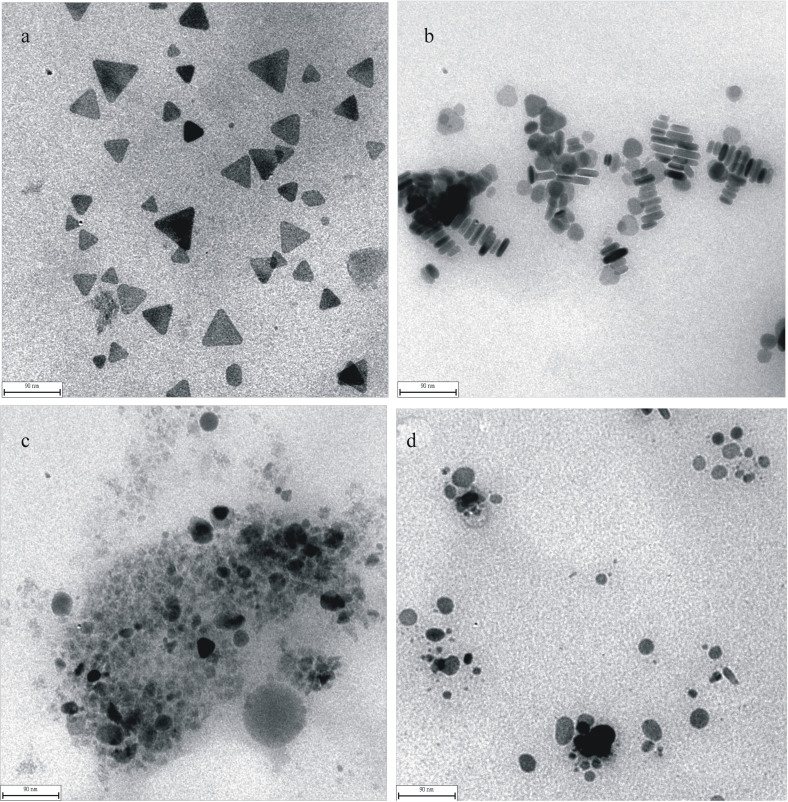
TEM of the nanosilve particles. (a): blue silver nanotriangle (scale bar 90 nm); (b): red silver nanorods (scale bar 90 nm); (c): 50 μmol/L AgNR + 3 mg/L BPO-pH 6.2, 65°C for 10 min; (d): 5.0 × 10^−5^ mol/L AgNR −5.0 × 10^−4^ mol/L NaCl-3 mg/L BPO-pH 6.2, 65°C for 10 min.

The transmission electron microscopy (TEM) of AgNPB (Figure 1a) shown that most are triangle nanosilver with the side length between 18–72 nm. Compared with AgNPB, the size of AgNRs was smaller with diameter of 9 nm and the length of 18–45 nm, in addition there are spherical nanosilvers (Figure 1b). The TEM of AgNR-BPO system (Figure 1c) shown that there is no AgNR, the unclear big particles are ascribed to the Ag-benzoic acid particles with low electron density and the clear small particles are the formed AgNPs. This indicated that the autocatalytic oxidation reaction take placed on the surface of AgNR and small AgNP particles formed when BPO was added. In the AgNR-BPO-NaCl system, on one hand, AgNR autocatalytic oxidized by BPO to generate Ag^+^. On the other hand the generated Ag^+^ on the surface of AgNR combined with Cl^−^ to form strongly hydrophobic AgCl particles with high electron density which made Ag atom was easier to be oxidized, and small AgNP generated with weak SPR absorption. Thus, there are some clear AgNP/AgCl particles in the system (Figure 1d).

### SPR absorption spectra

**Figure 2:**
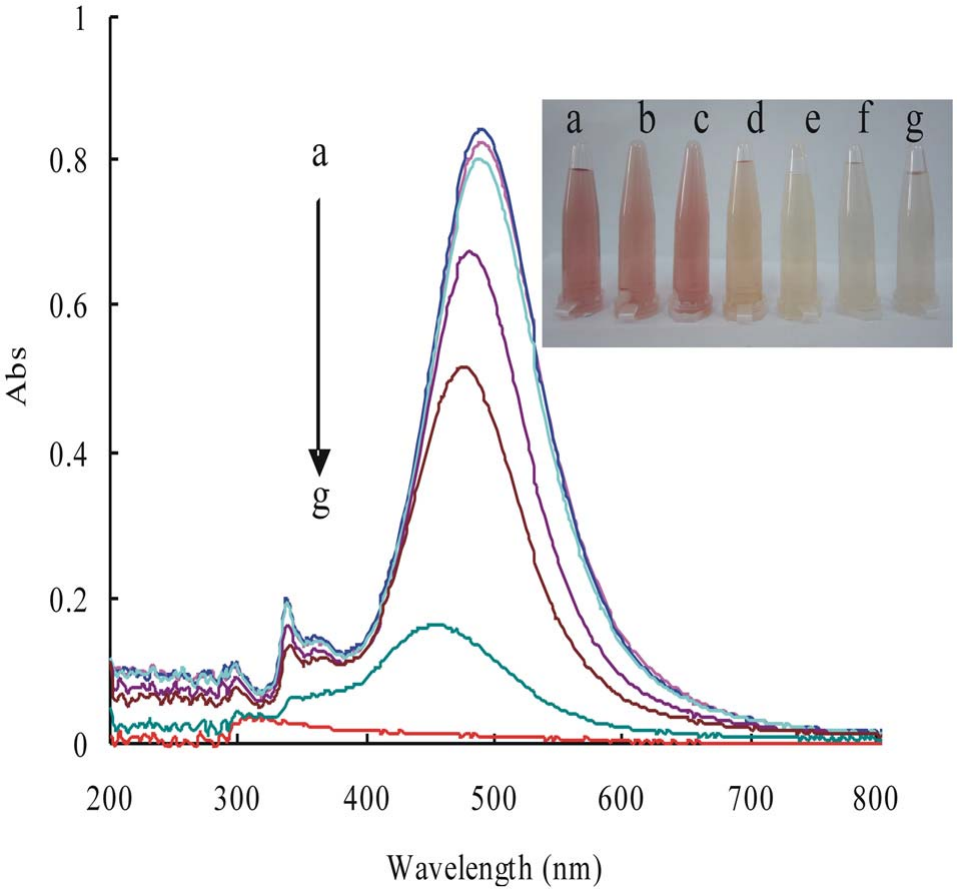
SPR absorption spectra of the AgNR-BPO system. (a): 50 μmol/L AgNR; (b): a + 0.04 mg/L BPO-pH 6.2; (c): a + 0.4 mg/L BPO; (d): a + 2 mg/L BPO; (e) a + 4 mg/L BPO; (f) a + 8 mg/L BPO; g a + 10 mg/L BPO, 65°C for 10 min.

The SPR characteristics of AgNP are mainly determined by the interaction between incident light and free electrons on the surface. SPR occurred when the wavelength of incident light coupled the vibration frequency of free electrons, and strong absorption peaks were exhibited in uv-vis spectra. The position of SPR peaks mainly depend on the size and shape of the nanoparticles, surface charge, environmental medium conditions and other factors. Spherical nanosilver had only one SPR peak at about 400 nm. AgNPB had three SPR peaks (Figure 1Sa). Silver nanorods had a transverse and a longitudinal SPR peak at 336 nm and 476 nm respectively (Figure 2Sa). In the presence of NaCl, the strongest SPR absorption peak of the three nanosilver systems decreased with the increasing of BPO concentration. Without NaCl, the strongest SPR absorption peak of the AgNR system decreased linearly with the increasing of BPO concentration (Figure 2). Thus, the system without NaCl can be used to detect BPO too.

### SPR-RS spectra

**Figure 3:**
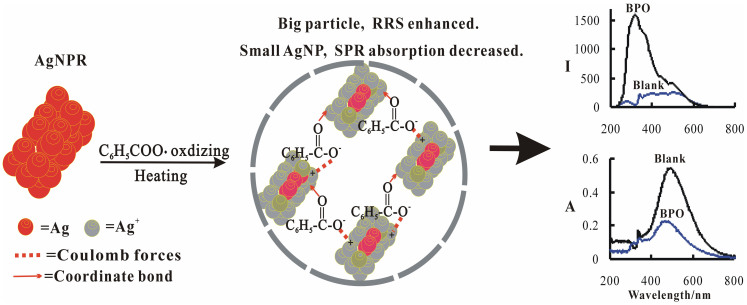
Principle of silver nanorods SPR methods to detect BPO.

**Figure 4:**
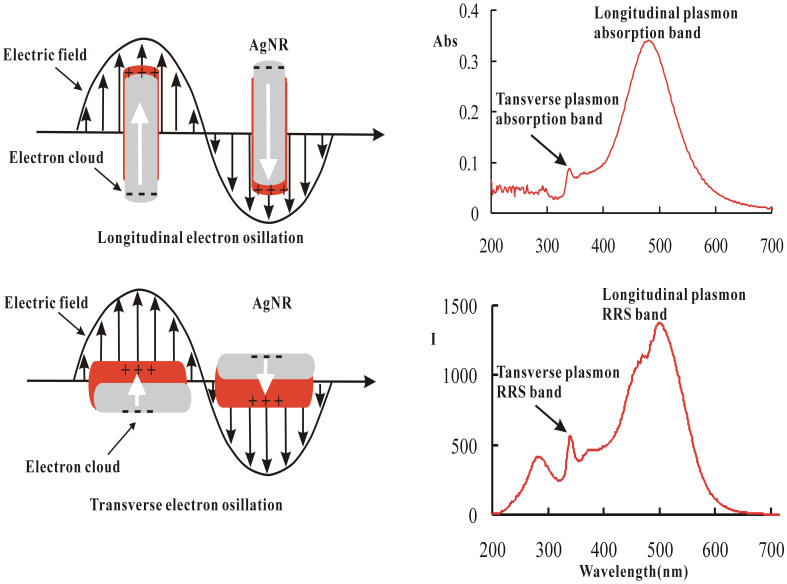
(A) Scheme of SPR electron oscillation for AgNRs, (B) SPR absorption and RRS bands of AgNRs. The longitudinal and transverse plasmon bands being ascribed to the electron oscillation along the long axis (Fig. 4A top) and the short axis (Fig. 4A below) of AgNR respectively.

When visible light irradiated the surface of the nanosilver, the light with the same wavelength as the resonant wavelength was absorbed that induced the surface electronic collective resonance to scatter photon outward. The scattering at resonant wavelength was the strongest since the absorbed photons at this wavelength was the most. Silver nanorods had two transverse and longitudinal SPR absorption bands, and its transverse and longitudinal SPR Rayleigh scattering bands were observed firstly at 342 nm and 495 nm (Figure 3Sa). The both are corresponded to each other and were in compliance with the law of conservation of energy. In general, the energy of Rayleigh scattering photon is less than that of photon absorbed due to the loss of the system energy. The silver nanorods had a Rayleigh scattering peak at near 280 nm that was due to the strong emission of fluorescence meter light source (Figure 3Sa). In the AgNR-BPO system, BPO oxidation ability enhanced with the increasing of BPO concentration that caused the AgNR concentration decreased, the SPR-RS peak decreased, and a new RS peak at 300 nm appeared. But the linear relationship between the Rayleigh scattering intensity and BPO concentration was not good. In the presence of NaCl, the SPR-RS peaks of the AgNPB and AgNR systems are linearly increased with the increase of BPO concentration (Figure 4S, 5S). For the AgNPB and AgNR systems, their strongest RRS peaks were 315 nm and 319 nm, respectively. Therefore, SPR-RS spectral methods can be used to detect BPO.

### SERS spectra of the AgNR-NaCl-VBB system

Solid nanosilver is commonly used as substrate in SERS research. It has high SERS activity but poor reproducibility, so it is difficult to use in SERS quantitative analysis. Stable nanosol, especially stably aggregated nanosol is very necessary and sufficient condition in SERS quantitative analysis^31^,^32^. In addition, stable particle with rough surface in solution is also used directly as SERS sol substrate in the quantitative analysis. In the SERS nanosol substrates,nanosilver sol is one of strongest SERS activity, and one of first choice. Non-aggregated colloidal silver nanoparticles prepared by heterogeneous nucleation of sodium citrate and hydrazine, were used to detect rhodamine 6 G by surface enhanced resonance Raman spectroscopy^33^. It has been found that nanoparticle aggregated sol exhibited stronger SERS active than its non-aggregated sol^31^,^34^,^35^,^36^,^37^,^38^. Silver nanorod has been used as SERS substrate^17^,^39^,^40^. A simple and sensitive approach using solvent-induced hot spot switch on silver nanorod enhanced Raman spectroscopy has been found, which can be used for detection and identification analyte molecules^17^. A single-step, multiplexed, homogeneous immunoassay platform was reported for sensitive detection of protein targets based on the high surface-enhanced Raman spectroscopy (SERS) signal enhancement by controlled assembly of gold nanorods^39^. The synthesis of AgNRs with different aspect ratios has been reported using a seed-mediated method and evaluation of their use for SERS^40^. In this article, the prepared red silver nanorod has good SPR properties and stability, and would be used in SERS quantitative analysis. The SERS properties of AgNR-NaCl system were studied using Victoria blue (VBB) as molecular probe, it had SERS peaks at 200 cm^−1^, 433 cm^−1^, 794 cm^−1^, 1169 cm^−1^, 1200 cm^−1^ and 1612 cm^−1^. Among them, the SERS peak intensity at 1612 cm^−1^ was largest and most sensitive. With the increase of BPO concentration, the SERS peak linearly decreased (Figure 6S). In absence of NaCl, the SERS peak shape of AgNR-VBB system was similar to that of AgNR-NaCl-VBB (Figure 7S), but the sensitivity was reduced greatly since there was no the aggregation of AgNRs. So, a SERS peak at 1612 cm^−1^ was chosen to detect BPO.

The effect of VBB concentration on SERS intensity was studied (Figure 8S). The system had the maximum of ΔI when the concentration of VBB was 0.4 μM. So a 0.4 μM VBB was chosen for use. And, the adding order of VBB had a great influence on the SERS intensity. The 1^st^ procedure was: AgNR-NaCl-BPO-VBB was added in turn and diluted to 2.0 mL before heating at 60°C water bath for 15 min. The 2^nd^ procedure was: VBB was added after water bath while the previous operation was the same as the first procedure. The result showed that the SERS intensity of the 1^st^ procedure was much bigger than that of the 2^nd^ procedure. So, the 1^st^ procedure was selected. The absorption spectrum of BPO-VBB was examined without AgNR. The result showed that the color and the absorption spectrum did not change since BPO couldn't oxidize VBB under the chosen conditions. The ΔI value in Figure 8Sa is larger than that in Figure 8Sb that did not caused by the BPO oxidation of VBB. The reasons may be that the VBB probes easily adsorbed on the AgNR to exhibited high SERS effect. After the oxidation reaction, the Ag-benzoic acid molecules may enwrap the AgNR surface and restrained the SERS activity. Thus, the SERS intensity decreased.

### Optimization of preparing conditions of AgNR

In the presence of surfactant PVP, a blue triangle nanosilver can be prepared by H_2_O_2_ and NaBH_4_ reduction^41^. Without PVP that could inhibited SERS activity, a blue triangle nanosilver can be also obtained by us^31^. Up to date, there is no repot about preparation of stable AgNR sol by heating the blue triangle nanosilver sol. Thus, the preparation conditions such as concentrations of sodium citrate, H_2_O_2_ and NaBH_4_ were studied. The results show that the blue triangle silver-nanoplate can be prepared by H_2_O_2_ and NaBH_4_ reduction of AgNO_3_ at room temperature, using 0.6–4.8 mmol/L sodium citrate without PVP (Figure 9Sa, 9Sc). The emergence of oxygen bubbles was due to H_2_O_2_ decomposition in the sol. The stability and reproducibility of blue nanosilver were not ideal, and residual H_2_O_2_ may affect the subsequent research. Stable light-blue nanosilver and AgNR can be prepared by heating the above two blue nanosilver sols with different concentration of sodium citrate, at 100°C water bath for 10 min (Figure9Sb, 9Sd). Five duplicate samples of AgNR were prepared (Figure 10S). The mean values of *I*_500 nm_ and A_500 nm_ of RRS spectra and absorption spectra were 1491 and 0.467 respectively, with RSD of 2.9% and 3.8%. This shown that the preparation of AgNR had good reproducibility. This new, simple, rapid, and practical preparation method for stable AgNR sol made it is easy to popularization and application in nanoanalysis.

### Optimization of the analytical conditions

The effect of the concentration of the both AgNPs on Δ*I* was studied (Figure 11S). When the concentration of AgNR or AgNPB was 5.0 × 10^−5^ mol/L, both the two systems had the maximum of Δ*I*. So a 5.0 × 10^−5^ mol/L AgNR and AgNPB were chosen. The effect of NaCl concentration on Δ*I* of the two systems was studied. For AgNR or AgNPB system, the Δ*I* value was maximal when the NaCl concentration was 0.5 mM or 0.625 mM (Figure 12S). The effect of pH (4.9–6.6) on Δ*I* was studied. The effect of pH on the Δ*I* was not great, the Δ*I* value was maximal for the both systems when the pH was 6.2. So pH 6.2 citric acid-sodium citrate buffer solution was chosen. The effect of reaction temperature on Δ*I* was studied (Figure 13S). The Δ*I* of AgNR-NaCl-BPO systems increased with the reaction temperature increased in the range of 20–60°C, while the Δ*I* decreased after 60°C. So a 60°C was chosen for the systems. The Δ*I* of AgNPB-NaCl-BPO system increased with the reaction temperature increased in the range of 20–80°C, while the Δ*I* decreased after 80°C. So 80°C was chosen for the system. The effect of reaction time on Δ*I* was studied (Figure 14S). The Δ*I* of AgNPB-NaCl-BPO and AgNR-NaCl-BPO systems reached the maximum when the reaction time is 10 min and 15 min respectively. So a 10 min and 15 min were chosen for the two systems.

### Analytical feature and application

According to the procedure, the RRS intensity (*I*) and SPR absorption (*A*) of different BPO concentration were measured, and the working curve between Δ*I* or Δ*A* and BPO concentration were drawn. The result showed that AgNR-NaCl-BPO RRS method was much sensitive, with a 0.005 mg/L of detection limit. The linear range was 0.01–3.5 mg/L, with the regression equation of Δ*I* = 511c-41, and a correlation coefficient of 0.9898 (Figure 15S). The linear range of AgNR-NaCl-BPO SPR system was 0.05–5 mg/L, with the regression equation of Δ*A* = 0.131c-0.017, a correlation coefficient of 0.9941, and a detection limit of 0.03 mg/L (Figure 16S). The linear range of AgNR-BPO SPR system was 0.04–10 mg/L, with the regression equation of Δ*A* = 0.0843c + 0.0078, a correlation coefficient of 0.9975, and a detection limit of 0.02 mg/L (Figure 17S). In addition, a SERS method for the quantitative analysis of BPO was established by chosen VBB as molecular probes and AgNR as substrate (Figure 18S). The linear range was 0.4–2 mg/L BPO, and the SERS method was less sensitive and simple than the AgNR SPR method. Among them, the AgNR-NaCl-BPO RRS system was the most sensitive and was chosen for use, while the AgNR-BPO SPR system was simple, less reagent used and stable. The relative standard deviation of five determinations of 0.10, 0.50 and 1.5 mg/L BPO is 5.0, 4.2 and 3.8% respectively, that showed the AgNR-NaCl-BPO RRS method is accuracy. Compared to reported methods for BPO (Table 1S), the AgNR-NaCl-BPO RRS method is one of the most sensitive. According to the procedure, the effect of co-existence substances on the AgNR-NaCl-BPO RRS method was studied, with a relative error of less than ±10%. Results showed that 100 times of ClO_3_^−^, Ca^2+^ and MoO_4_^2−^, 50 times of SeO_3_^2−^ and BrO_3_^−^, 10 times of H_2_O_2_, Fe^3+^ and Cu^2+^, 1 time of VO_3_^−^ did not interfere with the determination of BPO. This showed that this method has good selectivity. A 1.00 g flour sample was extracted by ultrasonic with 10 mL ethanol before high-speed centrifuging. A certain amount of supernatant fluid was taken to detect the BPO content according to the procedure. The analytical results are listed in Table 2S, and the recovery was in the range of 96–110%.

## Discussion

### Principle of silver nanorods SPR methods to detect BPO

BPO was unstable and formed strong oxidant benzoyl oxide free radical (C_6_H_5_COO·) under the condition of 60°C^42^. Ag atoms on the surface of silver nanorods can be oxidized by C_6_H_5_COO· to produce [Ag^+^]. Then, [Ag^+^] combined with benzoic acid to generate hydrophobic [Ag^+^]-benzoic acid complexes and formed larger particles through coulomb forces and coordinate bond (Figure 3). In addition, [Ag^+^] on the surface of silver nanorods combined with Cl^−^ to form strong hydrophobic AgCl^31^ in the presence of Cl^−^, and formed larger AgNR/AgCl particles. More silver nanorods were oxidized with more BPO was added, and more large size hydrophobic particles were formed. So the SPR absorption decreased linearly due to AgNR decreasing, while the RRS intensity increased due to big particles increasing. Accordingly, new SPR absorption and RRS methods for BPO have been proposed.

### The relationship between the SPR absorption and RRS of AgNR

Metal nanoparticles, especially nanogold and nanosilver, have novel optical properties and are different from the metal block. These are closely related to the SPR on the surface of nanoparticles^43^. In general, the presence of the SPR can lead to strong light absorption and Rayleigh scattering, and depends on the size and shape of particles, environmental medium and material composition, etc. However, rare SPR-RS peak was reported^31^, the main reason is that the RS strongly depend on the excited light intensity of fluorescence spectrometer. As we know, spherical nanogold or nanosilver had only one SPR absorption band^44^,^45^,^46^. Silver nanorods had two SPR absorption bands, including the transverse band at 338 nm and the longitudinal band at 480 nm (Figure 19S). Correspondingly, there had both transverse and longitudinal SPR-RS bands at 340 nm and 500 nm respectively. The transverse SPR-RS band was very close to the transverse SPR absorption band, while the wavelength of longitudinal SPR-RS band had a difference of 20 nm from that of longitudinal SPR absorption band. These were related to the uneven distribution of emission intensity of the light source. According to the principle of surface plasma electron oscillation of nanorods (Figure 4), nanorods SPR absorption caused SPR-RS. That is, 340 nm and 500 nm of Rayleigh scattering peaks were transverse and longitudinal SPR-RS peaks of silver nanorods respectively.

### Principle of the SERS enhancement mechanism of aggregated nanorods

In SERS mechanism, significant electromagnetic enhancement can originate from the individual nanoparticles in solution, a dramatic increase in SERS is achieved when the nanoparticles are positioned in close proximity to each other^47^. At present, the SERS enhancement of silver nanorod sol is relative scare. The nanorod is in same negative and cathodic charge that formed surface plasma (SP), and the nanorods disorderly and dispersedly exists in sol. Upon addition of NaCl, it caused the silver nanorod aggregations with very strong SERS signal. A SP aggregated-nanorod grating mechanism was developed to explain the SERS enhancement (Figure 20S). The SERS probe molecules of VBB may absorb on the surface of nanorods that produced Raman scattering signal. The total SERS signal is weak because the nanorods are independent and arrange disorderly. After addition of NaCl, the independent nanorods as plasma were aggregated orderly to aggregations that like grating platform, the nanorods are coupling by the NaCl molecules, the distance between the two nanorods is a constant that control by the salt, those nanorods form a quasi nano-grating, in which the Raman scattering photons take place diffraction with very strong SERRS signal.

### The formation of AgNR from AgNPB

**Figure 5:**
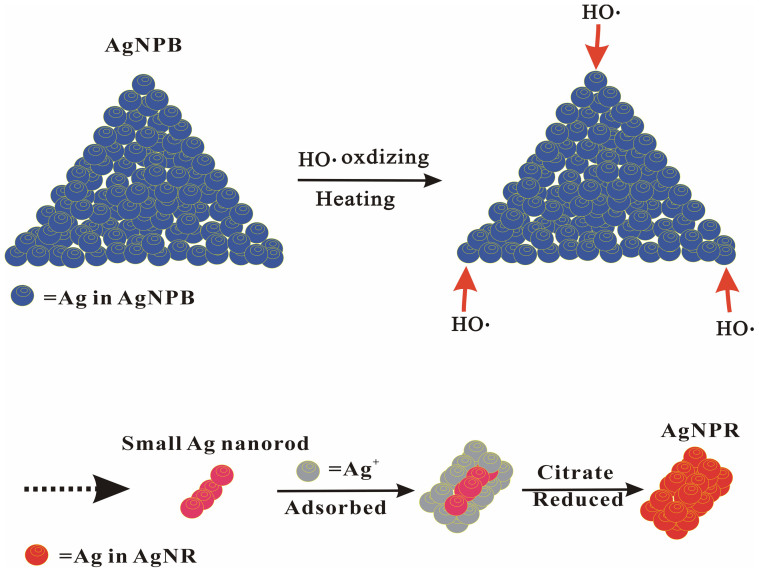
The formation of AgNR from AgNPB.

In the presence of reducing agent H_2_O_2_ and stabilizer sodium citrate, micro-amount of strong reducing agent NaBH_4_ quickly reduced Ag^+^ to small AgNP crystal nucleus at room temperature. The excess Ag^+^ adsorbed on the surface of the crystal nucleus as nanocatalyst was reduced by H_2_O_2_ to deposite on the surface to form blue triangular nanosilver^31^. The rest of H_2_O_2_ in AgNPB solution can be catalyzed by highly active AgNPs to form hydroxyl radicals (HO·) under the boiling bath. Then HO· oxidized the active Ag atoms on the edge of the triangle nanosilve to form small size red silver nanorods until the H_2_O_2_ was consumed. The oxidized Ag^+^ was also adsorbed on the surface of small silver nanorods, and was reduced by citrate to form stable red silver nanorod sol at 100°C (Figure 5).

## Methods

### Apparatus and reagents

A model of F-7000 fluorescence spectrophotometer (Hitachi Company, Japan), a model of TU-1901 double beams spectrophotometer (Puxi Tongyong Apparatus Limited Company, Beijing), and a model of JEM–2010 transmission electron microscope (Japanese electronics co., Ltd, Japan) were used. A 800 μg/mL BPO stored solution was prepared by dissolving in ethanol. A pH6.2 citric acid-sodium citrate buffer solution was prepared.

A 1.0 × 10^−4^ mol/L blue triangle nanosilver (AgNPB) was prepared as follows: a 47 mL water, 500 μL 1.0 × 10^−2^ mol/L AgNO_3_, 3.0 mL 6.0 × 10^−2^ mol/L sodium citrate, 120 μL 30% H_2_O_2_, 200 μL 0.1 mol/L NaBH_4_ were added into a triangle flask in turn with stirring to obtain AgNPB sol. Then, the AgNPB solution was heated at 100°C water bath for 10 min after it changed to red to obtain a 1.0 × 10^−4^ mol/L AgNR solution.

### Procedure

A 1.0 mL 1.0 × 10^−4^ mol/L AgNR solution, 20 μL 0.05 mol/L NaCl, 50 μL pH6.2 citric acid-sodium citrate buffer solution and a certain amount of BPO solution were added into a 5 mL calibrated tube in turn, then diluted to 2 mL and mixed well. The mixture was heated at 60°C water bath for 15 min before it was cooled to room temperature. The RRS spectra were recorded by a fluorescence spectrophotometer with synchronous scanning (*λ*_ex_-*λ*_em_ = Δ*λ* = 0). A blank (*I_0_*) without BPO was recorded and the value of Δ*I = I* − *I_0_* was calculated. The absorption values were recorded at 500 nm. A blank (*A*_0_) without BPO was recorded and the value of Δ*A* = *A*_0_-*A* was calculated.

## Author Contributions

* G.Q. and Z.L. contributed equally to this work. G.Q., Z.L. and Y.H. performed the experiment and prepared Fig. 1–4. G.Q., Z.L. and A.H. wrote the main manuscript text. Z.L., A.H., G.Q., Y.H., X.H. and Q.Y. contributed to the discussion and measurement analysis. All authors contributed to the preparation of the manuscript and reviewed the manuscript.

## Supplementary Material

Supplementary Information Supplementary Info File #1
