# Supplementary material for: A new silver nanorod SPR probe for detection of trace benzoyl peroxide
Source: Sci Rep. 2014 Jun 17;4:5323. doi: 10.1038/srep05323 (PMC4060507; doi:10.1038/srep05323)
Supplement: Supplementary Information — Supplementary Info File #1 [file srep05323-s1.doc]

**A new silver nanorod SPR probe for detection of trace benzoyl peroxide**

Zhiliang Jiang**^*^**, Guiqing Wen, Yanghe Luo, Xinghui Zhang, Qingye Liu, Aihui Liang**^*^**

(Key Laboratory of Ecology of Rare and Endangered Species and Environmental Protection of Ministry Education, Guangxi Normal University, Guilin 541004, China)

**Key words:** silver nanorod; stable nanosol; SPR; Rayleigh scattering; SERS; BPO; detection.

a

b

c

d

e

A

f

g

Wavelength/nm

Figure 1S Absorption spectra of the AgNPB-NaCl-BPO system

a: 5.0×10^-5^ mol/L AgNPB -6.25×10^-4^ mol/L NaCl-pH 6.2; b:a-0.4µg/mL BPO; c: a-1.6µg/mL BPO; d:a-3.2µg/mLBPO; e:a-4.0µg/mL BPO; f:a-6.4µg/mL BPO; g:a-7.2µg/mL BPO.


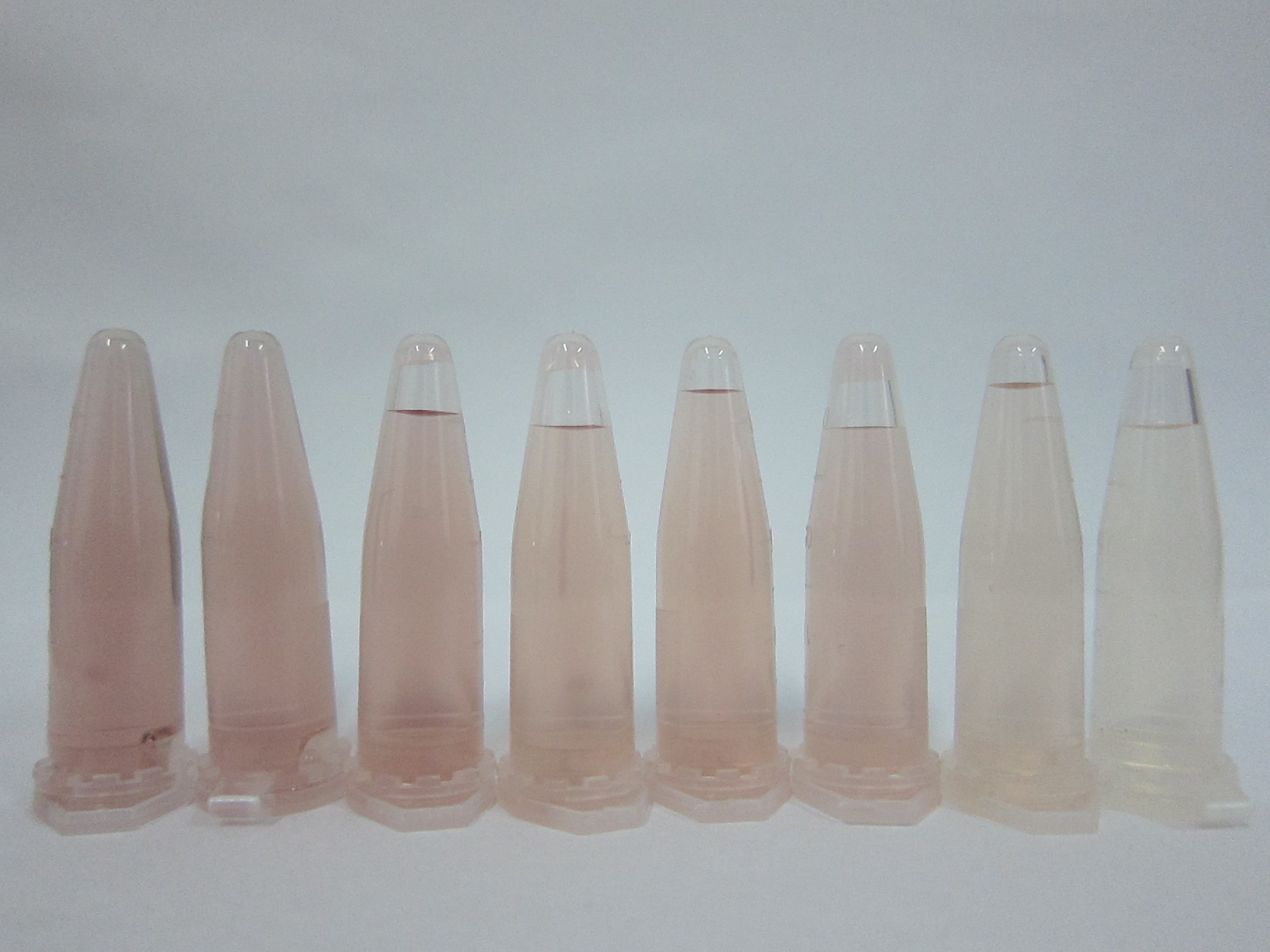


A

a

b

c

d

e

f

g

h

a b c d e f g h

Wavelength/nm

Figure 2S Absorption spectra of the AgNR-NaCl-BPO system

a: 5.0×10^-5^ mol/L AgNR -5.0×10^-4^ mol/L NaCl-pH 6.2; b:a-0.5µg/mLBPO; c: a-1.5µg/mLBPO; d:a-2.0µg/mLBPO; e:a-2.5µg/mLBPO; f:a-3.0µg/mLBPO; g:a-3.5µg/mLBPO; h:a-4.0µg/mLBPO.

a

b

c

d

e

f

Figure 3S RRS spectra of the AgNR-BPO system

a: 50μmol/L AgNR; b:a+0.4 mg/L BPO-pH 6.2; c:a+2 mg/L BPO; d: a+ 4 mg/L BPO; e: a+8 mg/L BPO; f: a+10mg/L BPO.

f

e

d

c

b

a

I

Wavelength/nm

Figure 4S RRS spectra of the AgNPB-NaCl-BPO system

a: 5.0×10^-5^ mol/L AgNPB-6.25×10^-4^ mol/L NaCl-pH 6.2; b:a-0.8mg/L BPO; c: a-1.6mg/L BPO; d:a-2.4mg/L BPO; e:a-3.2mg/L BPO; f:a-4.0mg/L BPO.

Wavelength/nm

I

h

g

f

e

d

c

b

a

Figure 5S RRS spectra of the AgNR-NaCl-BPO system

a: 5.0×10^-5^ mol/L AgNR -5.0×10^-4^ mol/L NaCl-pH 6.2; b:a-0.5µg/mLBPO; c: a-1.0µg/mLBPO; d:a-1.5µg/mLBPO; e:a-2.0µg/mLBPO; f:a-2.5µg/mLBPO; g:a-3.0µg/mLBPO; h:a-3.5µg/mLBPO.

Raman shift/cm^-1^

a

b

c

d

e

I

Figure 6S SERS spectra of the AgNR-NaCl–BPO-VBB system

a: 5.0×10^-5^ mol/L AgNR -5.0×10^-4^ mol/L NaCl-4.0×10^-7^ mol/L VBB-pH 6.2; b:a-0.4µg/mLBPO; c: a-0.8µg/mL BPO; d:a-1.5µg/mLBPO; e:a-2.0µg/mLBPO.

Raman shift/cm^-1^

a

b

c

d

I

Figure 7S SERS spectra of the AgNR-BPO-VBB system

a: 5.0×10^-5^ mol/L AgNR -4.0×10^-7^ mol/L VBB-pH 6.2; b:a-1.0µg/mLBPO; c: a-2.0µg/mLBPO; d:a-3.0µg/mLBPO.

ΔI

a

b

*µ*M VBB

Figure 8S Effect of VBB concentration on ΔI

5.0×10^-5^ mol/L AgNR-5.0×10^-4^ mol/L NaCl-4.0×10^-7^ mol/L VBB-0.4µg/mLBPO-pH 6.2

a: VBB was added before reaction; b: VBB was added after reaction.

**
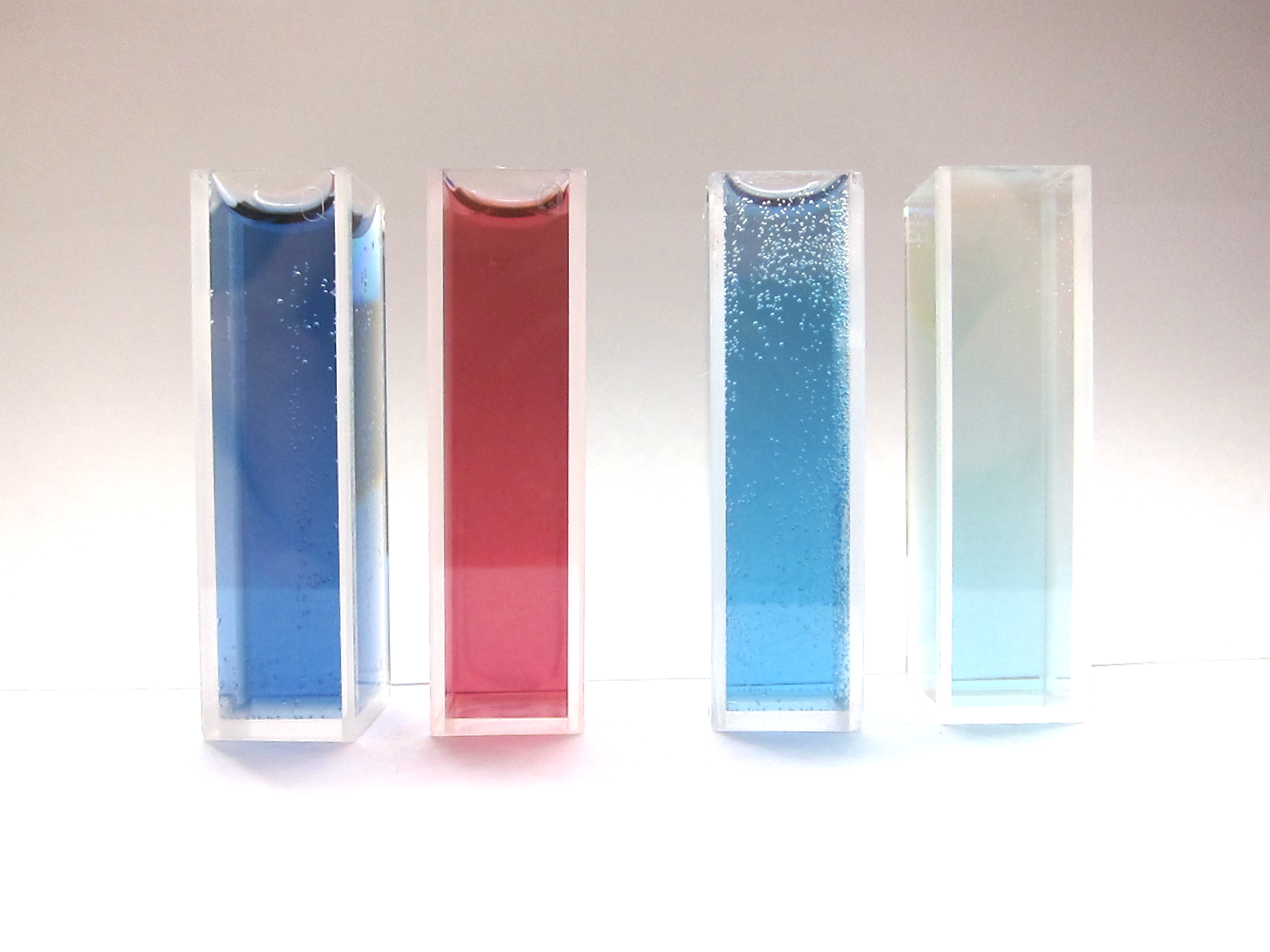
**

a b c d

Figure 9S Colors of the four AgNP sols

a: 0.1 mmol/L AgNO_3_-3.6mM sodium citrate -0.072% H_2_O_2_-0.4 mM NaBH_4_;b: a after heating at 100℃ water bath for 10min；c: 0.1 mmol/L AgNO_3_-0.6mM sodium citrate -0.072% H_2_O_2_-0.4 mM NaBH_4_; d: c after heating at 100℃ water bath for 10min.

I

A

Wavelength (nm)

Figure 10S RRS and absorption spectra of AgNR (n=5)

25μmol/L AgNR.

ΔI

**a**

**b**

µM AgNP

Figure 11S Effect of AgNP concentration on Δ*I*

a: AgNR-5.0×10^-4^ mol/L NaCl-4.0µg/mL BPO-pH 6.2; b: AgNPB-6.25×10^-4^ mol/L NaCl-4.0µg/mL BPO-pH 6.2.

ΔI

**a**

**b**

mM NaCl

Figure 12S Effect of NaCl concentration on Δ*I* of AgNR and AgNPB system

a: 5.0×10^-5^ mol/L AgNR-4.0µg/mL BPO-pH 6.2; b: 5.0×10^-5^ mol/L AgNPB-4.0µg/mL BPO-pH 6.2.

**a**

**b**

ΔI

T/℃

Figure 13S Effect of reaction temperature on Δ*I*

a: 5.0×10^-5^ mol/L AgNR-5.0×10^-4^ mol/L NaCl-4.0µg/mL BPO-pH 6.2; b: 5.0×10^-5^ mol/L AgNPB-5.0×10^-4^ mol/L NaCl-4.0µg/mL BPO-pH 6.2.

ΔI

**a**

**b**

t/min

Figure 14S Effect of reaction time on Δ*I*

a: 5.0×10^-5^ mol/L AgNPB-5.0×10^-4^ mol/L NaCl-4.0µg/mL BPO-pH 6.2; b: 5.0×10^-5^ mol/L AgNR-5.0×10^-4^ mol/L NaCl-4.0µg/mL BPO-pH 6.2.

ΔI

a

b

mg/L BPO

Figure 15S Working curve for AgNP-NaCl RRS method

a: 5.0×10^-5^ mol/L AgNR-5.0×10^-4^ mol/L NaCl-pH 6.2; b: 5.0×10^-5^ mol/L AgNPB-6.25×10^-4^ mol/L NaCl-pH 6.2.

a

b

ΔA

mg/L BPO

Figure 16S Working curve of SPR method of AgNP-NaCl system

a: 5.0×10^-5^ mol/L AgNR-5.0×10^-4^ mol/L NaCl-pH 6.2; b: 5.0×10^-5^ mol/L AgNPB-6.25×10^-4^ mol/L NaCl-pH 6.2.

Figure 17S Working curve of SPR method of AgNR-BPO system

50μmol/L AgNR-pH 6.2

ΔI

ΔI

a

b

µg/mL BPO

Figure 18S Working curve of SERS method of AgNP-NaCl system

a. 5.0×10^-5^ mol/L AgNR -5.0×10^-4^ mol/L NaCl-4.0×10^-7^ mol/L VBB-pH 6.2 ; b. 5.0×10^-5^ mol/L AgNR -4.0×10^-7^ mol/L VBB-pH 6.2.

A

B

a

b

c

d

Figure 19S Extinction spectra (A) and RRS spectra (B) of AgNR

d

c

b

a

T%

a: 5μmol/L AgNRs; b:12.5μmol/L AgNRs; c: 25μmol/L AgNRs; d:50μmol/L AgNRs.


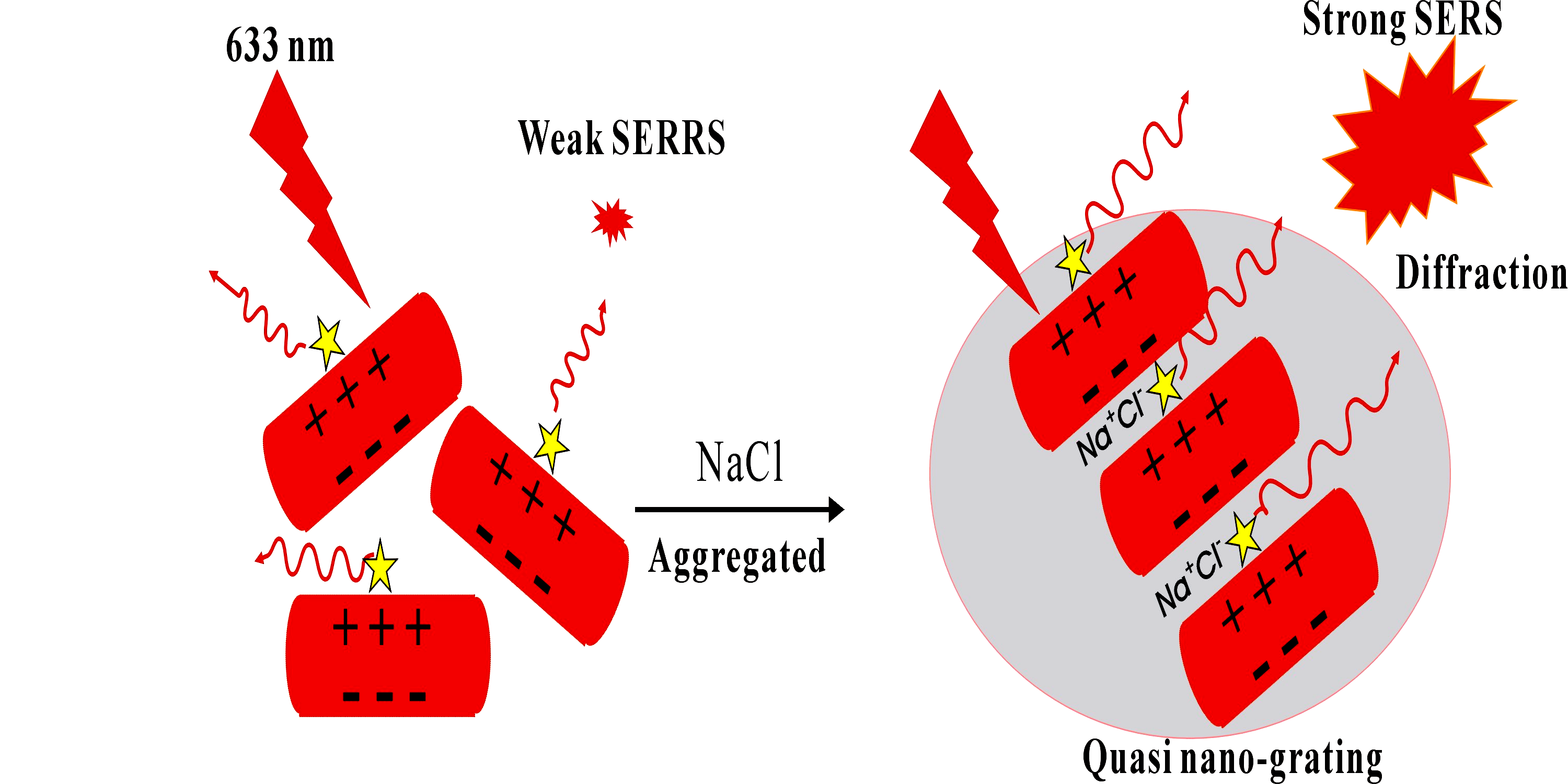


**Figure 20S** Principle of the SERS enhancement mechanism of aggregated nanorods

**Table 1S Analytical performance of some reported methods for BPO**

| Method | Principle | LR(µg/mL) | DL(µg/mL) | Comments | Ref. |
| --- | --- | --- | --- | --- | --- |
| Fluore-  scence | BPO oxidizes Fe^2+^ into Fe^3+^ that induces the opening of the spirolactam ring of N-methoxy rhodamine-6G spirolactam, switching on fluorescence of the detection system. | 0.2-3.2 | 0.06 | Sensitive | 23 |
| Chemilum-inescene | The determination of benzoyl peroxide was based on a capillary microliter order droplet sample jection–luminol chemiluminescence system. | 0.0005-1 | 0.00014 | Highly sensitive, but complicated. | 24 |
| Electro-  chemical | Carbon paste electrode contained fibers of coconut fruit, which are very rich in peroxidase enzymes naturally immobilized on its structure. | 1110-12210 | 555 | Low sensitivity. | 25 |
| Chromato-graphy | BPO was determined as benzoic acid after preceding reduction by potassium iodide. | 0.5-30 | 0.29 | Low sensitivity. | 26 |
| Fluoresce-nce | BPO oxidized the probe to form strong fluorescence | 0.12-6.3 | 0.06 | Selective. | 27 |
| RRS | BPO oxidized AgNR to form big particles with strong RRS effect. | 0.01-3.5 | 0.005 | Highly sensitive, simple. | This method |

**Table 2S Analytical results of flour** samples (n=3)

| Sample | BPO in the tube  /(μg.mL^-1^) | Added  /(μg.mL^-1^) | Total found  /(μg.mL^-1^) | Recovery /% | Content  /*μ*g.g^-1^ |
| --- | --- | --- | --- | --- | --- |
| 1 | 1.16 | 0.5 | 1.64 | 96 | 237 |
| 2 | 1.20 | 0.5 | 1.75 | 110 | 245 |
| 3 | 1.22 | 1.0 | 2.29 | 107 | 249 |
| 4 | 1.15 | 1.0 | 2.14 | 99 | 235 |
